# Characterization of in vivo chemoresistant human hepatocellular carcinoma cells with transendothelial differentiation capacities

**DOI:** 10.1186/1471-2407-13-176

**Published:** 2013-04-02

**Authors:** Christian Marfels, Miriam Hoehn, Ernst Wagner, Michael Günther

**Affiliations:** 1 Pharmaceutical Biotechnology, Department of Pharmacy, Ludwig-Maximilians-Universität, Butenandtstrasse 5-13, D-81377, Munich, Germany

**Keywords:** Cyclophosphamide, Chemoresistance, Tumor plasticity, Cancer stemness

## Abstract

**Background:**

Chemotherapeutic treatment of hepatocellular carcinoma often leads to chemoresistance during therapy or upon relapse of tumors. For the development of better treatments a better understanding of biochemical changes in the resistant tumors is needed. In this study, we focus on the characterization of *in vivo* chemoresistant human hepatocellular carcinoma HUH-REISO established from a metronomically cyclophosphamide (CPA) treated HUH7 xenograft model.

**Methods:**

SCID mice bearing subcutaneous HUH7 tumors were treated i.p. with 75 mg/kg CPA every six days. Tumors were evaluated by immunohistochemistry, a functional blood-flow Hoechst dye assay, and qRT-PCR for ALDH-1, Notch-1, Notch-3, HES-1, Thy-1, Oct-4, Sox-2 and Nanog mRNA levels. Cell lines of these tumors were analyzed by qRT-PCR and in endothelial transdifferentiation studies on matrigel.

**Results:**

HUH-REISO cells, although slightly more sensitive against activated CPA *in vitro* than parental HUH-7 cells, fully retained their *in vivo* CPA chemoresistance upon xenografting into SCID mice. Histochemical analysis of HUH-REISO tumors in comparison to parental HUH-7 cells and passaged HUH-PAS cells (*in vivo* passaged without chemotherapeutic pressure) revealed significant changes in host vascularization of tumors and especially in expression of the tumor-derived human endothelial marker gene PECAM-1/CD31 in HUH-REISO. In transdifferentiation studies with limited oxygen and metabolite diffusion, followed by a matrigel assay, only the chemoresistant HUH-REISO cells exhibited tube formation potential and expression of human endothelial markers ICAM-2 and PECAM-1/CD31. A comparative study on stemness and plasticity markers revealed upregulation of Thy-1, Oct-4, Sox-2 and Nanog in resistant xenografts. Under therapeutic pressure by CPA, tumors of HUH-PAS and HUH-REISO displayed regulations in Notch-1 and Notch-3 expression.

**Conclusions:**

Chemoresistance of HUH-REISO was not manifested under standard *in vitro* but under *in vivo* conditions. HUH-REISO cells showed increased pluripotent capacities and the ability of transdifferentiation to endothelial like cells *in vitro* and *in vivo*. These cells expressed typical endothelial surface marker and functionality. Although the mechanism behind chemoresistance of HUH-REISO and involvement of plasticity remains to be clarified, we hypothesize that the observed Notch regulations and upregulation of stemness genes in resistant xenografts are involved in the observed cell plasticity.

## Background

Hepatocellular carcinoma (HCC) is the fifth most common malignancy worldwide [1]. Moreover, its incidence increases due to hepatitis B and C viral infections. Therefore, HCC is in the focus of several treatment studies. Compared to other solid tumors, HCC is characterized by high levels of vascularization. The status of angiogenesis correlates with cancer progression and prognosis. Therefore, antiangiogenic strategies are suggested for treatment of HCC [2] due to survival advantages, as revealed in recent studies [3,4]. In addition, the usage of preclinical antiangiogenic, metronomic regimen of cyclophosphamide (CPA) revealed encouraging results in terms of tumor growth suppression and survival in an *in vivo* rat model of hepatocellular carcinoma [5]. The metronomic treatment regimen is characterized by significantly reduced side effects, compared to conventional maximum tolerated chemotherapy administration and by antitumoral activity in respect to its antiangiogenic properties. The metronomic treatment regimes target preferentially genetic stable tumor vessel endothelial cells and thus, the development of resistance against the therapy should be avoided [6,7]. However, several studies point towards the induction of *in vivo* chemoresistance mechanisms that let tumors escape from metronomic CPA therapy [8-10].

In this study, we investigated changes in transcription factors, controlling plasticity and stemness of tumor cells in an *in vivo* chemoresistance HCC xenograft mouse model. Resistant HCC xenografts were generated by metronomically scheduled CPA treatment in SCID mice, resulting in resistant tumor outgrowth after an initial chemoresponsive phase of 10 weeks. Histological analysis revealed significant changes in tissue organization and blood flow. Re-xenografted tumors from HUH-REISO cell culture manifested immediate chemoresistance, lacking an initial response phase. In order to detect gene expression associated with the chemoresistance and its development, expression levels of Notch-1 and downstream HES-1, Notch-3, Thy-1, Oct-4, Sox-2 and Nanog were determined in *in vivo* passaged control xenografts and in their resistant counterparts with and without therapeutic pressure. Furthermore, several aspects of cell differentiation were traceable in specialized *in vitro* models, mimicking features of environmental properties of solid tumors.

## Methods

### Cell culture

Cell culture media, antibiotics, fetal bovine serum (FBS) and trypsin/EDTA solution were purchased from Invitrogen GmbH (Karlsruhe, Germany). Human hepatoma cells (HUH-7) (JCRB0403) were cultured in a mixture of Ham’s-F12 and Dulbecco’s modified Eagle’s medium (DMEM) in a ratio of 1:1 supplemented with 10% FBS. Cells were grown at 37°C in 5% CO_2_ in a humidified atmosphere. HUH-7 cells were cultured without antibiotics for at least 3–4 passages before tumor cell implantation and were harvested just as reaching approx. 70% confluency.

### *In vivo* animal model

Male SCID mice (CB17/lcr-PrkdcSCID/Crl) (8–10 weeks) were housed in individually vented cages under specific pathogen free conditions with a 12 h day/night cycle and with food and water ad libitum. HUH-7 cells were cultured as described above. The number of 106 HUH-7 cells in 100 μl PBS was injected subcutaneously with a 25 G needle (Braun, Melsungen, Germany) into the flank of SCID mice. The animals were checked regularly for tumor progression. The moment that tumor volume reached the size of at least 10 mm3, tumor progression was monitored using a digital measuring slide (Digi-Met, Preisser, Gammertingen). Each measurement consisted of three diameters, length (a), width (b), and height (c). Tumor volume was calculated by the formula *a* × *b* × *c* × *π*/6 (with a, b and c indicating the three diameters and π/6 as correction factor for tumor shape). Tumor volume doubling time was calculated with *TVDT* = *ln*2*x*(*t*2 − *t*1)/*ln*[*V*(*t*2)/*V*(*t*1)]. All animal experiments were performed with 6 animals per group. All animal procedures were approved and controlled by animal experiments ethical committee of Regierung von Oberbayern, District Government of Upper Bavaria, Germany and carried out according to the guidelines of the German law of protection of animal life.

### Isolation of tumor cells

**Figure 1:**
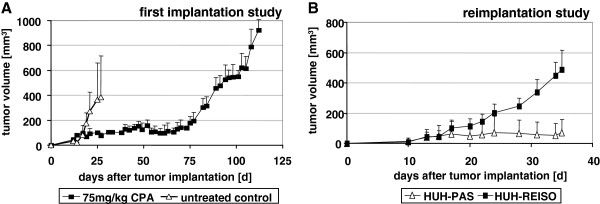
***In vivo *chemoresistance. **(**A**) Subcutaneous human HUH-7 tumors were established in SCID mice by injection of 5×10^6^ HUH-7 cells into the flanks of animals (n=6). CPA treatment was started on day 12 after tumor implantation with 75 mg/kg CPA every sixth days. Metronomically scheduled CPA treatment resulted in significant tumor growth delay. Tumor volume of treated mice was constant up to day 75 after tumor cell implantation, whereas tumors in the control group exhibited a tumor volume doubling time of 2.5 days. Around day 75 after tumor cell implantation tumor volume began to increase in the CPA treated group, despite ongoing treatment with a tumor doubling time of 3.5 days. (**B**) Cells isolated from resistant CPA treated tumors (HUH-REISO) were cultured *in vitro* and, after several passages, reimplanted in SCID mice (n=6) again, with CPA therapy (75mg/kg every sixth days) starting at day 10 after cell implantation. A cell line established from *in vivo* passaged tumor cells (HUH-PAS) served as control. Tumors derived from HUH-REISO cells revealed a tumor volume doubling time (under therapy) of 4.5 days, whereas tumor growth in the control group was not evident within the observed time.

For isolation of tumor cells, mice were sacrificed at the first therapy endpoint (see Figure 1 and Additional file 1: Table S1) with CO_2_. Skin was cleaned and sanitized with isopropanol (70% in water v/v), followed by drying under sterile conditions. Tumors were collected and immediately immerged in a 1:1 mixture of Ham’s-F12 and Dulbecco’s modified Eagle’s medium (DMEM), supplemented with 10%FBS and 2% penicillin/streptomycin (Biochrom, Berlin, Germany). Tumor tissue was reduced to small sections under sterile conditions. Pieces were chosen randomly from all areas of the tumor. This procedure was repeated until the tumor tissue was homogenized. The obtained homogenized cell suspension was diluted with fresh penicillin/streptomycin containing Ham’s-F12 and DMEM 1:1. The tumor cell containing suspension was transferred to tissue 6-well-plates (TPP, Trasadingen, Switzerland) and incubated under standard conditions (37°C, 5% CO2) in a humidified atmosphere for 2–3 days. Just as cells attached to the bottom of the plate, medium was replaced every second day, until cells reached a confluence of about 70%. Obtained cell lines (HUH-PAS, HUH-REISO) were defined in Additional file 1: Table S1. Reimplantation studies were performed by injection of 10^6^ HUH-7 tumor cells at a passage number below 10.

### Chemotherapy

Cyclophosphamide (CPA) (Sigma, Taufkirchen, Germany) was solved in PBS (10 mg/ml) and applied intraperitoneally. 75 mg/kg CPA solution was administered with a 25 G needle (Braun, Melsungen, Germany). The application of the CPA solution was carried out every 6 days. A single dose of each application was based on animal body weight. Toleration of CPA treatment was monitored by regular measurement of body weight. The vehicle group (PBS) and the drug treatment group (CPA dissolved in PBS) were housed separately.

### HE stain of tumors

Cryosections were fixed with 4% paraformaldehyde and stained with haematoxilin (Sigma, St. Louis, USA) for 30 min. After washing with PBS and aqua dest., sections were incubated with eosin (1:100 in aqua dest.) (Sigma, St. Louis, USA) for 4 min. Afterwards, sections were washed with aqua dest., embedded with PBS and analyzed by transmission light microscopy at the Axiovert 200 microscope (Zeiss, Jena, Germany).

### Immunohistochemistry

For immunohistology, tumors were embedded in tissue freezing medium (Jung, Nussloch, Germany). They were cut into sections of 5–10 μm thickness with a cryomicrotome (Leica CM 3050 s, Wetzlar, Germany) at − 20°C. Sections were transferred to a microscope slide, tissue freezing medium was removed and tissue was fixed with 4% paraformaldehyde (in PBS). Afterwards, sections were rehydrated and washed with blocking solution (PBS containing 5% FBS) prior to antibody incubation. Antibodies, which were used for the stains, are listed in Additional file 2: Table S2. All primary antibodies were diluted 1:200 in blocking solution. After incubation for 12 h at 4°C in humidified atmosphere, sections were washed repeatedly with blocking solution followed by secondary antibody staining. Secondary antibodies were diluted 1:400 in blocking solution and sections were incubated for 2 h at room temperature in humidified atmosphere. Sections were washed with blocking solution repeatedly, before fluorescence analysis at the Axiovert 200 microscope (Zeiss, Jena, Germany) using appropriate filter sets.

### Agarose overlay method

Cells were seeded in 6-well plates 24 h before addition of the agarose overlay. Culture medium was removed and replaced with 1 ml medium containing 0.6% (w/v) agarose (Invitrogen, Carlsbad, USA). The agarose-containing medium was obtained by stepwise dilution of complete medium with melted agarose (5% agarose in medium without supplementations, w/v). Before applying the agarose-containing medium to the seeded cells, the medium was allowed to cool to 37°C. After solidification, 2 ml of complete culture medium without agarose was added to the cells.

### Spheroid growth in agarose gel

To avoid cell growth in a two dimensional way, cells were seeded into an agarose gel. Therefore, cells were harvested as soon as reaching approx. 70% confluency. The gel was prepared by mixing low melting agarose from Cambrex BioScience (Rockland, USA) with medium in a percentage of 10% (w/v). After autoclaving, the gel was diluted with medium to 1.2%. Afterwards gel and single cell suspension were mixed and 5,000 cells in 1 ml of 0.6% gel were seeded in a 24 well plate (TPP, Trasadingen, Switzerland). Grown spheroids were counted after 42 days. Pictures were taken with an Infinity 2 camera and Infinity capture software (both: Lumenera corporation, Ottawa, Canada).

### *In vitro* matrigel angiogenesis assay

Matrigel was purchased from BD (Franklin Lakes, USA). The coating procedure was done as described in the BD guidelines for thin gel layers. Matrigel was thawed over night at 4°C. After short homogenization of the gel by pipetting with cooled tips, 10 μl of matrigel were added to each well of a μ-slide angiogenesis uncoated chamber (ibidi, München, Germany). The slide was incubated for 30 minutes at 37°C and afterwards cells were seeded on the gel layer in an amount of 50,000 cells in 50 μl of cell culture medium per well. Cells were grown at 37°C in 5% CO2 in a humidified atmosphere and observed with an axiovert 200 microscope (Zeiss, Jena, Germany). Pictures were taken after 24 hours with an Infinity 2 camera and Infinity capture software (both: Lumenera corporation, Ottawa, Canada). Image analysis was performed with the analysis software from S.Core (Hoehenkirchen, Germany).

### qRT-PCR

Marker gene analysis was done by quantitative polymerase chain reaction. Therefore, pairs of primer were designed with the universal probe library of Roche and purchased from Matabion (Martinsried, Germany). All pairs of primer, which were used for qRT-PCR, are listed in Additional file 3: Table S3. Total RNA of *in vivo* and *in vitro* samples were purified by using two different methods. For the *in vitro* angiogenesis assay samples, 10^6^ cells were seeded on a thin layer of matrigel (BD, Franklin Lakes, USA) in a six well plate. Total RNA of cells from one well was purified by phenol-chloroform extraction using peqGOLD TriFast kit (Peqlab, Erlangen, Germany). Preparation of *in vivo* RNA-Samples was done with a NucleoSpin RNA II kit (Macherey-Nagel, Düren, Germany) using 30 mg of tumor tissue. 5 μg of purified RNA were applied for cDNA production. For each qRT-PCR 25 ng cDNA were used for amplification and all samples were measured in duplicates. After an activation cycle with 90°C for 10 Minutes, 45 cycles were performed with a 10 seconds denaturation step at 95°C, 30 seconds of annealing at 60°C and polymerase extension for 1 second at 72°C. The PCR runs were performed with Light Cycler 480 (Roche, Mannheim, Germany). For detection, the corresponding probe out of the universal probe library (Roche, Mannheim, Germany) was applied. Fold changes were calculated with the ΔΔCt- method. As GAPDH and Act-B showed stable unregulated expression status over all tested tumor samples in initial experiments, GAPDH was used as reference gene for measurements of Oct-4, Thy-1, and ALDH-1 and Act-B for measurements of HES-1, Notch-3, Notch-1, Nanog, Sox-2, PECAM-1/CD31, and ICAM-2. The ready to use Universal Probe Library (UPL) reference gene assays for GAPDH and ACT-B (Roche Diagnostics, Mannheim, Germany) were applied.

### *In vitro* CPA sensitivity

*In vitro* sensitivity was assayed by measuring the DNA content of a cell population composed of 25% of CPA activating X39 cells mixed with either HUH-wt, or HUH-REISO cells, followed by CPA treatment. Generation of CYP2B1 transgene expressing X39 cells is described previously [11]. In total, 1500 cells per well were plated in 48-well plates. Twenty-four hours after seeding, the culture medium was removed and replaced by either 200 μl of fresh medium or by fresh cell culture medium containing CPA at the indicated concentrations. Treated cells and controls were incubated for 5 days in a humidified atmosphere containing 5% CO_2_ at 37°C. DNA contents were assayed after Hoechst 33258 incorporation, as previously described [11]. Briefly, cells were lysed with Millipore water followed by a freeze–thaw cycle. Cell lysis buffer (1 mM Tris-EDTA, pH 7.4, 200 mM NaCl) containing 0.2 ng/ml Hoechst 33258 was applied to each well, followed by another freeze–thaw cycle. The DNA content was measured by quantifying fluorescence with a plate reader (Tecan, Grödig, Austria) equipped with filters for excitation at 360 nm and emission at 465 nm. Relative DNA content was calculated using the ratio of DNA content treated /DNA content untreated cell culture.

### Statistical analysis

U-Test (Mann–Whitney) analysis was performed with WinStat for Exel to proof statistical significance in all cases. * stands for p≤0.05, ** for p≤0.01.

## Results

### HUH7 tumors under metronomic CPA therapy *in vivo*

Male and female SCID mice bearing subcutaneously implanted human HUH7 tumors were treated with metronomically scheduled CPA (75 mg/kg every 6 days). CPA treatment was started on day 12 after tumor cell implantation, just as tumors reached an average volume of 32 mm^3^. Metronomically scheduled CPA treatment resulted in a significant tumor growth delay. The tumor volume of treated mice was constant at around 100 mm^3^ up to day 75 after tumor cell implantation, whereas tumors in the control group exhibited a tumor volume doubling time of 2.5 days. Around day 75 after tumor cell implantation, tumor volume began to increase in the CPA treated group, with a tumor doubling time of 3.5 days, despite ongoing treatment (Figure 1A). Metronomically scheduled CPA therapy was well tolerated, indicated by a constant animal body weight up to day 85 after tumor cell implantation (data not shown). Further CPA treatment resulted in significant loss of body weight, observed in all CPA treated animals (data not shown). Treatment was stopped and the mice were sacrificed as soon as the average body weight loss reached 20%. At this therapy endpoint, tumors were collected and subjected to macroscopical and histological analyses. Furthermore, tumor cells were extracted from viable tumor tissue for characterization and cell culture experiments. To establish appropriate control cells (HUH-PAS), HUH7 were grown in male and female SCID mice, without exposure to therapy. As soon as tumors reached about 300 to 400 mm^3^, viable cells were extracted from tumor tissue. Reisolated cells (HUH-REISO and HUH-PAS) exhibited the same morphology in comparison to the parental HUH7 cells (HUH-wt) and were identified by human EGF-Receptor staining for their human origin (Additional file 4: Figure S1).

### Influence of CPA therapy on tumor macroscopic appearance, tumor histology and functional blood flow

**Figure 2:**
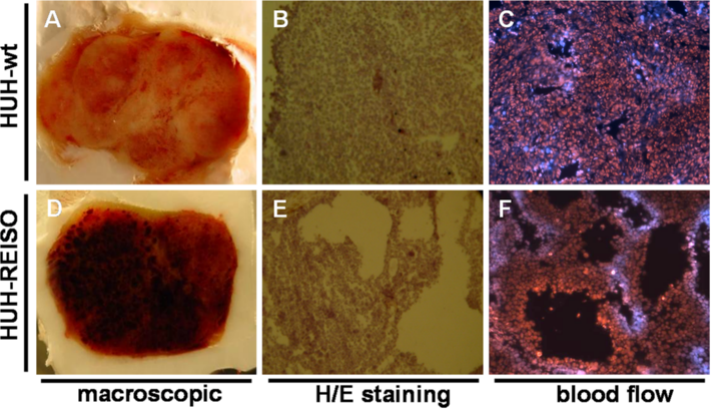
**Macroscopic appearance, tumor histology and functional blood flow. **(**A-C**) Untreated parental tumor (HUH-wt) and (**D-E**) CPA treated *in vivo* resistant tumor at treatment endpoint (HUH-REISO), all collected at day 25 after tumor cell implantation. Cryosections (8 μm) were fixed with 4% paraformaldehyde (PFA) and subjected to H/E staining (**B**) untreated parental tumor (HUH-wt) and (**E**) *in vivo* resistant tumor at treatment endpoint (HUH-REISO). Functional blood flow was visualized by intravenous application of Hoechst 33258 dye (blue). (**C**) Untreated parental tumor (HUH-wt) and (**F**) *in vivo* resistant tumor at treatment endpoint (HUH-REISO).

Tumors at the first therapy endpoint were macroscopically assessed. The tumor tissue appeared dark and bloody (HUH-REISO) (Figure 2D), compared to the untreated control tumors (HUH-wt) (Figure 2A). For further characterization and evaluation of changes induced by *in vivo* passaging and CPA treatment, histological and immunohistological analyses were performed. Therefore, cryosections were stained with haematoxylin/eosin and analyzed by transmitted light microscopy. Tissue structure in the original HUH7 xenografts (established from HUH-wt) was compact and homogeneous (Figure 2B). In contrast, resistant tumors (HUH-REISO) exhibited an inhomogeneous, sponge-like structure with large cavities within the tumor tissue (Figure 2E). These cavities were identified as intratumoral blood lakes, due to the presence of erythrocytes. To verify this finding, *in vivo* tumor blood flow was visualized by systemically applied Hoechst 33258 dye as a tracer. Several cavities within the tumor tissue from resistant HUH-REISO tumors exhibited tracer fluorescence, indicating connection with the systemic blood supply (Figure 2F). For comparison, functional blood supply analysis was performed also for the original xenografted HUH7 tumors (HUH-wt, Figure 2C) and reimplanted treated HUH-PAS tumors (Additional file 5: Figure S3 A-C). These HUH-PAS control tumors showed a clear diminished functional blood flow.

### Immunohistochemical analysis of vascular structures in xenografts

**Figure 3:**
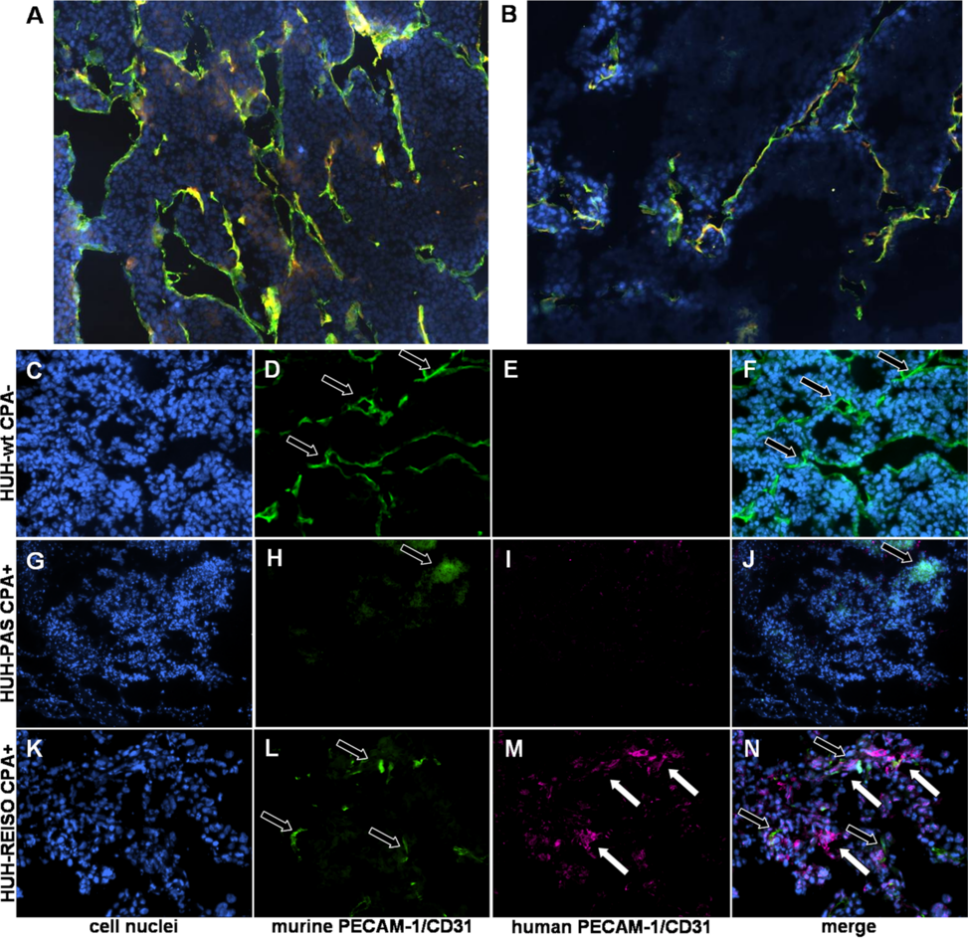
**Immunohistochemical analysis for vascular markers in HUH-7 tumors.** Cryosections (5 μm) of untreated control tumors (HUH-wt) (**A**) and CPA treated, chemoresistant tumors HUH-REISO (**B**) were fixed with 4% PFA and stained with antibodies for murine CD31/PECAM-1 (green) and laminin (yellow). Significant changes in the arrangement of laminin and CD31/PECAM-1 positive endothelial cells were detected in CPA treated tumors *versus* control tumors. Functional blood flow was visualized by intravenous application of Hoechst 33258 dye (blue). Additional immunohistochemically stained cryosections of untreated HUH-wt (**C-F**) tumors, and HUH-PAS (**G-J**) or HUH-REISO (**K-N**) tumors after two times CPA treatment are shown. Staining for murine CD31/PECAM-1 (D/H/L, green, highlighted with black arrows) and human CD31/PECAM-1 (E/I/M, magenta, highlighted with white arrows) and cell nuclei were counterstained with DAPI (C/G/K). Adjacent to signals from murine CD31/PECAM-1, significant human CD31/PECAM-1 expression was detected in (K-N, HUH-REISO) chemoresistant, CPA treated tumors, whereas human CD31/PECAM-1 expression was not detected in (C-F, HUH-wt and G-J, HUH-PAS) control tumors.

Immunohistochemical analysis of the vessel associated markers murine PECAM-1/CD31 and laminin showed an obvious shift from initial tumor vascularization (HUH-wt) (Figure 3A), as it is typical for HUH7 xenografts, to a tumor tissue with a decreased murine vessel density (HUH-REISO) (Figure 3B) at the therapy endpoint. Interestingly, functional blood flow, indicated by Hoechst tracer staining, was not closely correlated with immunohistochemically identified vessel structures (Figure 3B).

Regarding plasticity aspects, tumor tissue was stained for human, besides mouse, endothelial specific marker PECAM-1/CD31 (hPECAM-1 and mPECAM-1). Counter stain of cell nuclei with DAPI can be seen in Figure 3C (HUH-wt), 3G (HUH-PAS) and 3K (HUH-REISO). Control stains revealed no hPECAM-1 signal for the parental HUH7 xenografts (Figure 3E and F), whereas mPECAM-1 (mCD31) positive cells showed a large network of vessel (Figure 3D, highlighted with black arrows). Most interestingly, Figure 3M and merged 3N showed hPECAM-1 (hCD31) positive structures (highlighted with white arrows) in reimplanted resistant xenografts (HUH-REISO) in close neighbourhood to murine vascular structures (Figure 3L), indicating HCC plasticity towards the endothelial lineage. Control tumors of HUH-PAS, which were treated twice with CPA, showed rare positive signals for mPECAM-1 (Figure 3H) and no positive signal for hPECAM-1 (Figure 3I). Those HUH-PAS tumors had to be treated late, when they had reached an average volume of 254 mm^3^. At an earlier starting point of therapy of these chemoresponsive cells there would not be enough tumor material left for reliable analysis (Figure 1B).

### No evidence of acquired resistance *in vitro*

**Figure 4:**
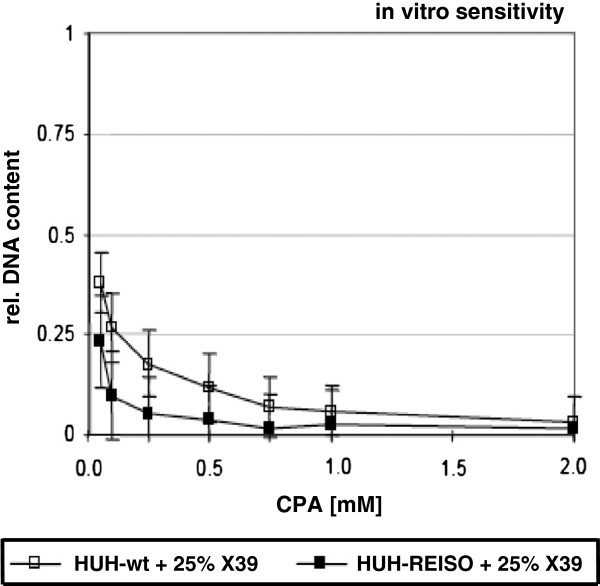
**Sensitivity of parental HUH-wt and isolated HUH-REISO cells towards *in situ *activated CPA.** Parental and isolated cells were treated with different concentrations of CPA for 3 days together with CPA activating cells X39. Proliferation was determined by measuring total DNA content per well. Control experiments were performed in the absence of CPA. Mean values ± SD of four measurements are shown. No significant differences were observed, nevertheless HUH-REISO cells showed higher sensitivity.

Original HUH7 cells (HUH-wt) and HUH-REISO tumor cells were treated in an *in vitro* co-culture model together with X39 cells, expressing the CYP450 transgene to convert CPA *in situ* into activated CPA [11]. As shown in Figure 4, the *in vivo* resistant HUH-REISO as well as the HUH-wt cells showed CPA concentration-dependent decrease in cell proliferation, indicating no manifestation of resistance in the *in vitro* setting. Interestingly, the *in vivo* resistant HUH-REISO displayed an insignificantly higher chemosensitivity.

### Resistance of reimplanted tumors towards metronomic CPA therapy *in vivo*

Reisolated HUH-REISO tumor cells and reisolated *in vivo* passaged (HUH-PAS) cells were implanted in the flank of SCID mice. On day 10 after tumor cell implantation, just as average tumor volume reached 14 mm^3^, mice were subjected to CPA treatment (75mg/kg, every 6 days). Tumor volume and body weight were measured regularly during the treatment. No growth retardation effect was detectable for xenografts established from HUH-REISO cells, in contrast to blocked growth of xenografts established from *in vivo* passaged cells (HUH-PAS) (Figure 1B). Resistant xenografts exhibited an average tumor volume doubling time of approximately 4.5 days. Metronomically scheduled CPA was again well tolerated, indicated by no significant loss in body weight (data not shown).

### Regulation of ALDH-1 expression in response to CPA therapy *in vivo*

**Figure 5:**
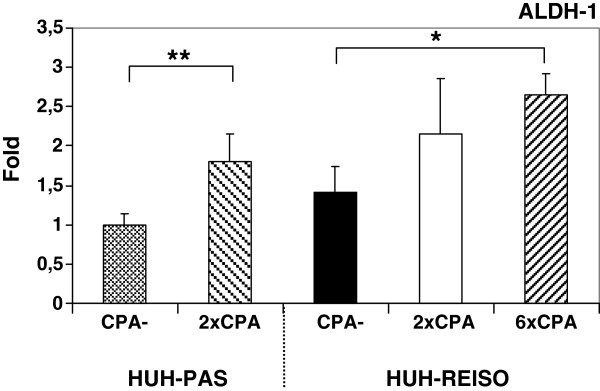
**Aldehyde dehydrogenase I (ALDH-1) expression levels.** Influence of CPA treatment on expression levels of ALDH-1 was determined in tumor tissue by qRT-PCR analysis after two treatments (HUH-PAS and HUH-REISO) and additional four treatments in the case of HUH-REISO (n=5). CPA therapy induced ALDH-1 expression in HUH-PAS (p=0.009) and in HUH-REISO (p=0.01) xenografts significantly. However, the ALDH-1 level was similar for passaged (HUH-PAS) and resistant (HUH-REISO) xenografts, independent of therapy. Statistic evaluation was performed using the Wilcoxon-Mann–Whitney test. P< 0.05 was considered as significant and indicated by *, p< 0.01 was indicated by **.

As *homo sapiens* aldehyde dehydrogenase 1 family member A1 (ALDH-1) is a known detoxification enzyme and inactivates CPA intermediates, expression levels were measured in HUH-REISO and in HUH-PAS during therapy. In absence of CPA pressure, only insignificant differences in expression levels were detectable in HUH-PAS and in HUH-REISO tumors (Figure 5). However, during therapy, expression levels of ALDH-1 increased in both xenograft types significantly after two treatments. In resistant tumors, ALDH-1 expression levels increased 2.5-fold after six treatments. With a p-value of 0.35, the ALDH-1 mRNA levels are not statistically different between HUH-PAS and HUH-REISO after the 2nd treatment (2×CPA).

### Expression profiles of Thy-1, Oct-4, Sox-2 and Nanog *in vivo*

**Figure 6:**
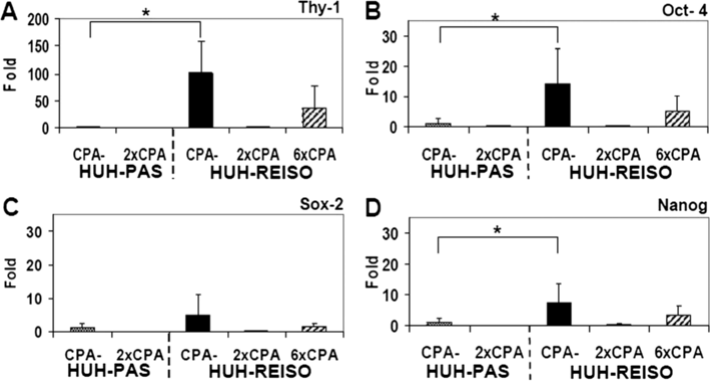
**Expression of the plasticity markers Thy-1, Oct-4, Sox-2 and Nanog in tumor tissue.** Influence of CPA treatment on expression levels of (**A**)Thy-1, (**B**) Oct-4, (**C**) Sox-2 and (**D**) Nanog was determined in tumor tissue by qRT-PCR analysis without (CPA-) and in tumor tissue after two (2×CPA+; HUH-PAS, HUH-REISO) and six (6×CPA+; HUH-REISO) treatments (n=5 for each column). CPA-sensitive HUH-PAS tumor would not survive a 6×CPA- long-term treatment in sufficient extent required for analysis. Statistic evaluation was performed using the Wilcoxon-Mann–Whitney test. P< 0.05 was considered as significant and indicated by *.

For characterization of stemness as a possible cause of tumor cell plasticity, the well established stemness markers Thy-1, Oct-4, Sox-2 and Nanog were analyzed, after total RNA extraction from tumor tissue. Expression analysis of untreated mice revealed that expression levels of Thy-1 (Figure 6A), Oct-4 (Figure 6B) and Nanog (Figure 6D) were significantly increased in resistant tumors. In contrast to tumors, which were grown from HUH-PAS cells, Sox-2 (Figure 6C) was not significantly increased in resistant tumors. In untreated resistant tumors, Thy-1 expression levels were about 100-fold (p=0.014) higher, Oct-4 expression levels were about 14-fold increased (p=0.027), Sox-2 expression levels were 5-fold (p=0.086) upregulated and expression levels for Nanog were detected to be increased about 7-fold (p=0.05), in comparison to tumors established from passaged tumor cells (Figure 6A-D). Notably, early after initiation of CPA treatment (two times CPA therapy) expression levels of Thy-1, Oct4, Sox2 and Nanog were found to be transiently decreased to low expression levels, indicating transient reduction of stemness (see discussion). Moreover, after long term treatment (6 times of CPA therapy), expression levels of all four pluripotency markers rose in the resistant tumors again.

### Expression profiles of Notch-1, Notch-3 and HES-1

**Figure 7:**
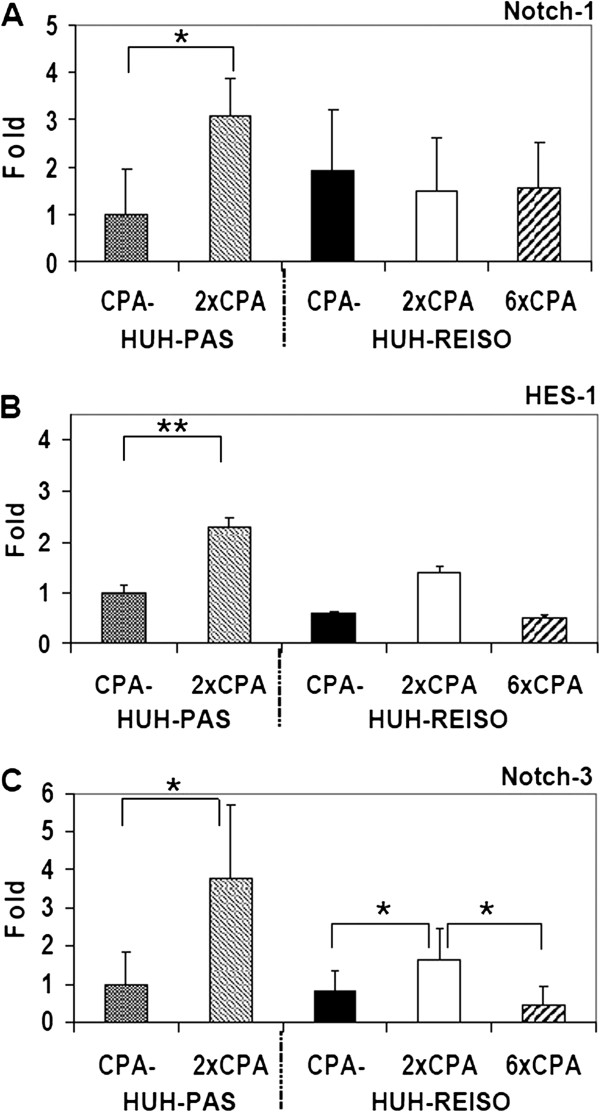
**Expression of Notch-1, Notch-3 and its downstream target HES-1 in tumor tissue.** Influence of CPA treatment on expression levels of Notch-3, Notch-1 and its downstream target HES-1 were determined in tumor tissue by qRT-PCR analysis before and after two treatments (HUH-PAS and HUH-REISO) and further four treatments in the case of HUH-REISO (n=5). (**A**) In contrast to significant induction of Notch-1 expression by two CPA therapies in passaged tumors (HUH-PAS), Notch-1 expression levels in chemoresistant tumors (HUH-REISO) remained not significantly altered even after further four CPA-treatments. Initial expression levels of Notch-1 were not significantly different. (**B**) HES-1 expression levels were detected to be significantly induced after two treatments for passaged (HUH-PAS) and chemoresistant tumors (HUH-REISO). Initial expression levels and expression levels after two CPA-treatments remained significantly low compared to tumors grown from *in vivo* passaged cells (HUH-PAS). After further four treatments, expression levels again reached initial HES-1 expression in chemoresistant tumors (HUH-REISO). (**C**) Notch-3 showed in both groups HUH-PAS (p=0.0247) and HUH-REISO (p=0.0446) significantly upregulated levels after two times of CPA therapy. After additional four CPA treatments, level of Notch-3 in HUH-REISO dropped back on base levels.

Passaged (HUH-PAS) and resistant (HUH-REISO) tumor bearing mice were treated by metronomic CPA therapy. Interestingly, Notch-1 expression (Figure 7A) was conversely regulated in comparison to Thy-1, Oct-4, Sox-2 and Nanog. Significant increase of Notch-1 (about 3-fold) expression was detected only in *in vivo* passaged tumors (p=0.028) after two times of CPA treatment. Regulation of Notch-1 in already resistant tumors was not observable. Even after six times of chemotherapy, Notch-1 expression levels stayed constant. However, expression of HES-1, a target gene of the Notch pathway, was upregulated after two CPA-treatments in passaged and in resistant tumors. In HUH-PAS tumors, levels of HES-1 were 2.3 fold higher (p=0.0090), in HUH-REISO tumors 2.4 fold higher (p=0.0143) (Figure 7B), if compared with the corresponding non-treated counterparts. After six treatments, HES-1 expression levels sank to the levels of untreated tumors. HES-1 expression levels were about 2-fold higher in *in vivo* passaged tumors, compared to *in vivo* resistant tumors, independent of the treatment. Congruent to Notch-1 regulation, significant increase of Notch-3 expression levels (about 3.7fold) were detected in *in vivo* passaged tumors (p=0.0247) after two times of CPA treatment (Figure 7C). In resistant tumors an increased level about 2fold (p=0.0446), which appeared after two treatments, disappeared after further four treatments.

### Anchorage independent growth of HUH-wt, HUH-PAS and HUH-REISO spheroids

**Figure 8:**
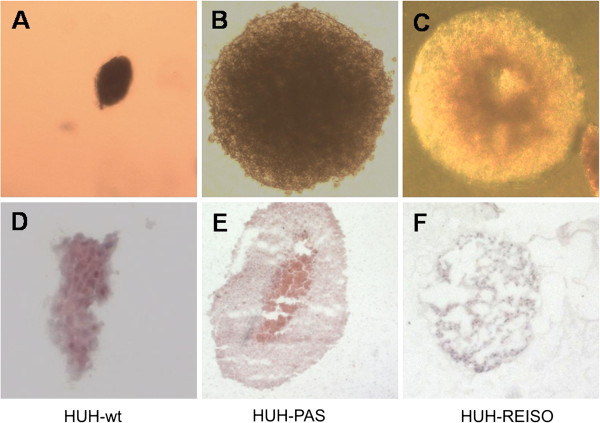
**Anchorage independent growth.** Multicellular spheroids grew from a single cell suspension of (**A**) parental HUH-7 cells (HUH-wt), (**B**) *in vivo* passaged HUH7 cells (HUH-PAS) and (**C**) chemoresistant cells (HUH-REISO) in low melting agarose for 42 days and pictures were taken under a phase contrast microscop. Spheroid tissue organization was detected by H/E staining of cryoslides. (**D**) Parental HUH-7 cells (HUH-wt), (**E**) *in vivo* passaged HUH7 cells (HUH-PAS) and (**F**) chemoresistant cells (HUH-REISO).

For characterization of cell dependency on essential matrix signalling, the capacity for anchorage-independent growth was tested by their ability to form colonies in soft agar. Multicellular spheroids were counted 42 days after embedding the single cell suspension (5000 cells/well) in solid medium. No significant differences between resistant and non-resistant tumor cells could be observed (data not shown), as only HUH-wt built far less spheroids than the other two cell lines. Nevertheless, the spheroids differed in their appearance. The parental HUH-wt cells (Figure 8A) and *in vivo* passaged cells (Figure 8B) built up very compact and homogeneous spheroids. In contrast, spheroids from the resistant tumor cells (Figure 8C) were characterized by cavities within the spheroids. These findings could be consolidated by HE staining of 10μm cryosections of spheroids. HUH-wt spheroids (Figure 8D) and HUH-PAS spheroids (Figure 8E) showed uniform and continuous tissue without cavities, whereas HUH-REISO spheroids (Figure 8F) presented a sponge-like structure inside the spheroids.

### Endothelial transdifferentiation *in vitro*

**Figure 9:**
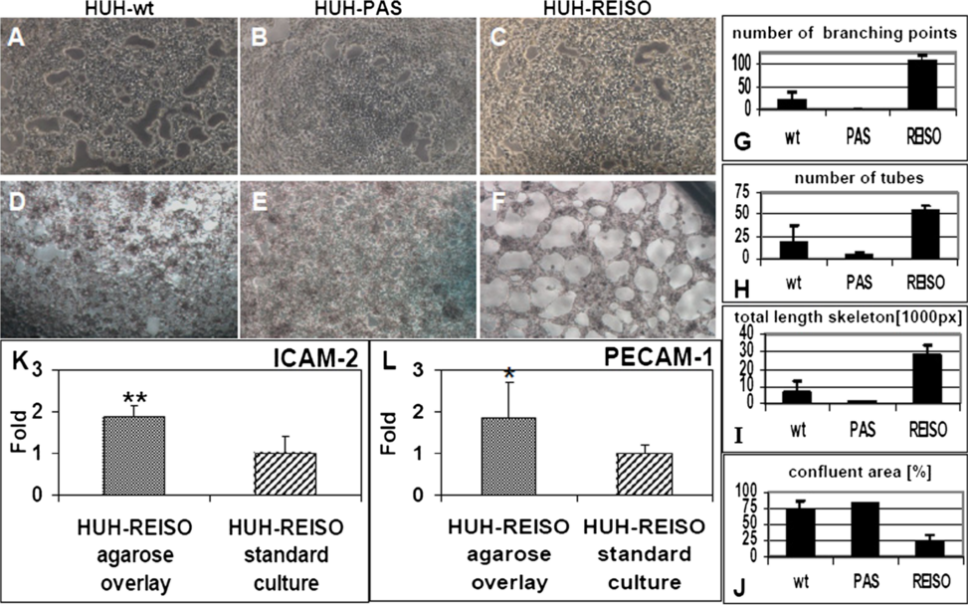
**Tube formation assay.** Tumor cells were seeded on matrigel after conventional cell culture (21% Oxygen / 37°C) or after a 42 days culture in a diffusion limited environment with reduced oxygen and nutrient supply. Pictures were taken 24h after plating. (**A**) Parental HUH7 (HUH-wt), (**B**) *in vivo* passaged (HUH-PAS) and (**C**) chemoresistant (HUH-REISO) cells derived from conventional cell culture did not show tube formation, whereas in the case of cells, derived from the diffusion limited environment, the (**F**) chemoresistant tumor cells (HUH-REISO) showed significant tube formation potential in the matrigel assay, in comparison to (**D**) HUH-wt and (**E**) HUH-PAS (n=5). Software based analysis of pictures revealed a significantly increased (**G**) number of branching points, (**H**) overall number of tubes and (**I**) total length skeleton in chemoresistant cells (HUH-REISO) in comparison to both control cell lines (HUH-wt and HUH-PAS). Consequently, (**J**) the total confluent area was significantly decreased for HUH-REISO (n=3). A qRT-PCR analysis, performed after the tube formation assay on the endothelial markers (**K**) ICAM-2 and (**L**) PECAM-1/CD31, revealed significantly increased expression levels for cells derived from the diffusion limited environment culture compared to conventional cultured cells, which did not show tube forming potential (n=6).

To evaluate the potential of tumor cells to transdifferentiate into an endothelial phenotype, a tube formation assay (Figure 9) was performed. At first, HUH-wt, HUH-PAS, and HUH-REISO cells were pre-cultured under conventional conditions (Figure 9A-C) or under a thin layer of solid medium (“agarose overlay”, Figure 9D-F) for six weeks. In such a diffusion controlled environment [11], supply with nutrients and oxygen and moreover, dilution of autocrine and paracrine factors is limited, compared to conventional cell culture systems. After the pre-culture, the six different cell culture groups were subjected to a conventional matrigel assay (Figure 9A-F). Only HUH-REISO cells, pre-cultured by agarose overlay, showed enough plasticity to form endothelial like tubes within 4h (data not shown). After 24h, the network was fully trained in this group (Figure 9F), whereas HUH-wt (Figure 9D) and HUH-PAS (Figure 9E) cells showed no striking tube formation. Moreover, tube formation was not detectable in all three tumor cell groups pre-cultured under conventional conditions (Figure 9A-C).

Quantification of tube formation in matrigel was performed via software based analysis (Additional file 6: Figure S2 and Figure 9G-J). Comparison of HUH-wt, HUH-PAS, and HUH-REISO (all pre-cultured by agarose overlay) revealed a far higher number of branching points (Figure 9G) and tubes (Figure 9H), a very extended length of skeleton (Figure 9I), and a decreased amount of confluent areas without tube formation (Figure 9J) for HUH-REISO cells.

Furthermore, expression levels of endothelial markers (CD31/PECAM-1, ICAM-2, VEGFR2, VE-cadherin and vWF) of agarose overlay HUH-REISO cells, showing positive tube formation, were compared to the corresponding standard culture HUH-REISO (Figure 9K-L). Expression levels were determined after the matrigel assay. In agarose overlay HUH-REISO cells, ICAM-2 (p=0.009) (Figure 9K), as well as CD31/PECAM-1 (p=0.028) (Figure 9L) were significantly upregulated. Expression of the other endothelial markers was not detectable in HUH-REISO under any culture condition (data not shown).

## Discussion

In the present study, acquired *in vivo* chemoresistance against metronomic cyclophosphamide (CPA) treatment was studied in a human hepatocellular carcinoma HUH7 xenograft mouse model. During treatment, a two phase development of tumor progression was observable: In the beginning of treatment (response phase), tumor progression was significantly decreased, indicated by constant tumor volume for about 75 days. In the following, second phase (escape phase), tumor volume increased with a tumor volume doubling time of 3.5 days, despite ongoing therapeutic intervention (Figure 1A). Viable tumor cells were extracted from resistant tumors (HUH-REISO), whereas control cells (HUH-PAS) were obtained from *in vivo* passaging HUH7 tumor cells without CPA treatment. Subsequently, HUH-PAS and HUH-REISO were characterized and identified in terms of cell morphology and representative human epidermal growth factor (EGF receptor) expression for their human origin (Additional file 4: Figure S1).

Interestingly, *in vivo* chemoresistant HUH-REISO did not manifest their drug resistant phenotype in a two-dimensional monolayer culture in presence of *in situ* activated CPA (Figure 4). In addition, significant changes in macroscopic appearance and tumor tissue organization of chemoresistant tumors indicate resistance mechanisms, which were only gainful in the *in vivo* situation. However, an endogenous imprinted component for *in vivo* chemoresistance was obvious, as the chemoresistant phenotype of isolated tumor cells was immediately manifested again after reimplantation and reapplied chemotherapy. In the reimplantation experiment, chemoresistance was manifested lacking the response phase. In contrast, HUH-PAS, which were only adjusted to the *in vivo* environment but not to the treatment, remained sensitive (Figure 1B) in a response phase. The qRT-PCR assay on *in vivo* samples revealed no significant difference in basal expression of the detoxification enzyme ALDH-1 (Figure 5), which converts aldophosphamide into carboxyphosphamide. ALDH-1 expression was detected to be significant upregulated *in vivo* during therapeutic pressure. However, the extent of upregulation was not significantly different in chemosensitive and chemoresistant tumors. The detected chemosensitivity of HUH-PAS exclude resistance by unspecific selection processes *in vivo*, which might change cellular properties independently of therapeutic pressure [12,13].

As metronomic CPA treatment is known to suppress tumor angiogenesis [6], functional blood flow analysis was performed, revealing blood flow in a part of the newly formed cavities of chemoresistant tumors (Figure 2F). In contrast to untreated animals, blood flow was not obligatory colocalized with immunohistochemical detected laminin and mouse PECAM-1/CD31 signal (Figure 3B). Further analysis on human vessel markers revealed within the tumor the presence of cells expressing human PECAM-1/CD31 (Figure 3M/N), indicating plasticity of the tumor cells and initiation of differentiation towards the endothelial lineage. This differentiation was observed only in HUH-REISO. In HUH-PAS the CPA therapy led to a deletion of murine vessels (Figure 3H), while the capacity of building new human vessels was not observed. As HUH-REISO showed no diminished blood-flow (Additional file 5: Figure S3) and also a supplementation of destroyed murine vessels by human endothelial cells, resistance is partly caused by a better blood-supplementation due to the higher plasticity of HUH-REISO. Better blood-supplementation leading to higher drug delivery into the tumor presumably requires also other detoxification mechanisms for the resistance. The higher levels of ALDH-1 in HUH-REISO (6×CPA, Figure 5) are consistent with this hypothesis. Therefore, further changes in the microenvironment and proteome of the resistant tumor cells remain to be examined in future work.

**Figure 10:**
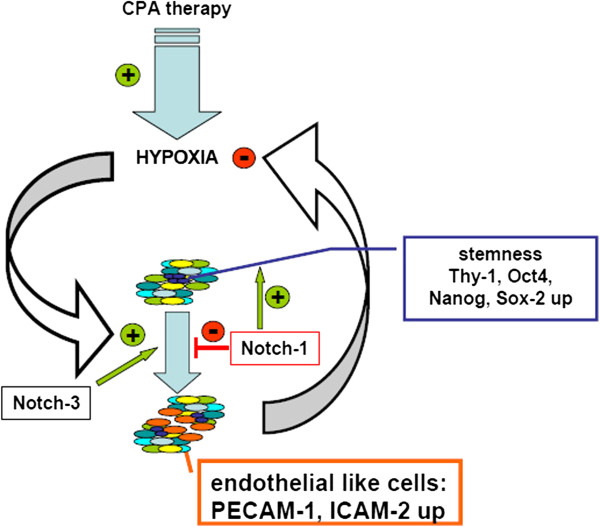
**Acquired chemoresistance towards metronomic CPA therapy – a hypothetical model of the equilibrium between stem cells and endothelial like cells in HUH-REISO.** During therapeutic pressure tumor cells acquire stepwise pluripotency. Reprogramming is associated with regulations of the Notch-pathway (Notch-1), resulting in enrichment of cells with increased expression levels of Thy-1, Oct-4, Sox-2 and Nanog. This pool of cells is the basis for increased adaptiveness of the tumor to therapy effects as hypoxia. Differentiation to a functional endothelial like phenotype, connected with tissue reorganization, counteracts antiangiogenic therapy. Finally, in the chemoresistant tumors an equilibrium between differentiation and self-renewal exists. In contrast to chemoresistant tumors, induction of Notch-3 expression in chemosensitive tumors leads to the induction of a differentiation process, which nearly flushes out the whole pool of pluripotent tumor cells.

Plasticity becomes often apparent in combination with the capacity of self-renewal and potential of anchorage independent growth, often assessed in spheroid building capacity assays [14,15]. In such an assay, no significant differences between resistant and non-resistant tumor cells could be observed (data not shown). In contrast to spheroids derived from HUH-wt (Figure 8A/D) or from *in vivo* passaged HUH-PAS cells (Figure 8B/E), spheroids formed from HUH-REISO cells revealed the formation of cavities and tubular structures (Figure 8C/F). This indicated cellular changes, which differed from the environmental conditioning of *in vivo* passaging. The consequent sponge-like growth (both in spheroids and in tumors) might also ensure an easier supplementation of HUH-REISO tissue with nutrients and oxygen than in compact HUH-PAS and HUH-wt tissue. Thus, for experiments HUH-PAS cells were used as an adequate control, to characterize development of chemoresistance *in vivo*. Taking the detected plasticity into account, HUH-PAS and HUH-REISO *in vivo* tumors were analyzed for expression profiles of markers, which are highly expressed in embryonic cells and described in recent papers about tumor initiating cells. Expression of Oct-4 plays a crucial role in maintaining pluripotency in stem cells [16]. Additionally, Sox-2 and Nanog expression contribute to plasticity, self-renewal and stemness [17]. Especially, in the context of HCC tumor stem cell research, Thy-1 expression is discussed as one crucial regulator of stemness and its upregulation is described in the context of chemoresistance [18]. In the absence of chemotherapy (−CPA, Figure 6A-D) *in vivo* qRT-PCR revealed significantly increased expression levels of Thy-1, Oct-4, Sox-2 and Nanog in the reimplanted chemoresistant HUH-REISO tumors compared with the *in vivo* passaged HUH-PAS control tumors. This indicates an inherent difference of the chemosensitive HUH-PAS and chemoresistent HUH-REISO tumors, with an enrichment of tumor cells with a reprogrammed, embryonic-like status for HUH-REISO. To monitor the response of HUH-REISO tumors towards CPA treatment with time, qRT-PCR analyses were performed also after 2× CPA and 6× CPA treatment. HUH-PAS tumors were analyzed in parallel, but for 2 treatments only and not the late time point (6× CPA), because of lack of tumor outgrowth on the one hand, or upon outgrowth conversion into a REISO-type resistant tumor on the other hand. At the earlier (2×CPA) timepoint resistance was not yet established for HUH-PAS (day 14, no tumor outgrowth, Figure 1B). *In vivo* passaging alone generated only few cells with pluripotent capacities (HUH-PAS, CPA-), but these cells differentiated very fast or got lost under therapeutic pressure (HUH-PAS, 2×CPA). In contrast, HUH-REISO generate tumor tissue containing a pluripotent subpopulation (Figure 6, -CPA). Upon CPA treatment HUH-REISO tumors continue to grow, but partly lost their pluripotent stem cell population during the acute response to the first two treatments (Figure 6, HUH-REISO, 2×CPA), presumably by differentiation. Chemoresistent tumors however were able to regenerate the pool of stem cells (Figure 6, HUH-REISO, 6xCPA) and reached a “steady-state” with stemness markers again. Based on these stemness marker results, recent papers [19-22] and our results regarding Notch-pathways, we propose the hypothesis shown in Figure 10.

In line with literature [19], the process of reprogramming was connected to induction of Notch-1 expression, as a first response to chemotherapeutic pressure (Figure 7A). Induction of Notch-1 leads to the expression of GIMAP5 [19], which is antiapoptotic and has potential to save cells. ALDH-1 was simultaneously induced (Figure 5) and led to cell survival under therapeutic pressure. Furthermore, induction of HES-1, which is a downstream target of Notch-1, was observable (Figure 7B). HES-1 belongs to the basic helix loop helix family of transcription factors and is described by Kageyama *et al.* as a crucial factor in many tissues to maintain the status of pluripotency [20]. Notch-1 and HES-1 were only upregulated in the response phase of therapy, indicating an initial key factor in this process. In the escape phase of already chemoresistant tumors, Notch-1 was steadily regulated under chemotherapeutic pressure, indicating that Notch-1 was essential for the preservation of the reprogrammed status, once Thy-1, Oct-4, Sox-2 and Nanog provides pluripotency and self-renewal.

Surprisingly, after two treatments with CPA, expression levels of Thy-1, Oct-4, Sox-2 and Nanog were decreased in HUH-REISO (Figure 6A-D), indicating a process of differentiation, which obviously antagonize further enrichment of this subpopulation at this point of treatment. This correlated with the induction of Notch-3 expression. The Notch-3 pathway is described as an important signalling pathway in the development of vascularization. N. Lawson *et al*. [21,22] showed the important role of Notch-3 in arterial cell fate during blood vessel development. However, after prolonged CPA treatment, expression levels of all pluripotency markers recovered. The initial expression profile of Notch-3 and the profile of Thy-1, Oct-4, Sox-2 and Nanog pointed to an adaptation process, as a response to acute chemotherapeutic pressure. Obviously, differentiation was repressed again after adaptation and the balance returned to enrichment of pluripotent cells. An important step to the *in vivo* chemoresistance in our model was therefore the development of a pool of cells, which reveal increased levels of pluripotency markers and obviously help to overcome chemotherapeutic pressure by initiation of differentiation (Figure 10). In contrast to chemoresistant tumors, the CPA therapy in chemosensitive tumors led to the induction of differentiation processes and cell death, which in the beginning of treatment nearly flushed out the whole pool of pluripotent tumor cells. Recovering of this pool took long time (growth delay phase Figure 1B) and led, beside other mechanisms, in the end to resistance (Figure 1A).

This differentiation process, resulting in the reorganization of tumor tissue, helped tumors to escape the metronomic treatment. To investigate the trigger, which caused differentiation, HUH-wt, HUH-PAS, and HUH-REISO cells, were pre-cultured in an *in vitro* cell culture system, which mimics several features of solid tumors under therapeutic pressure. In a following matrigel tube formation assay, usually performed for angiogenesis studies [23], HUH-REISO cells showed tube formation capacity (Figure 9F), whereas cells pre-cultured under conventional conditions showed no functionality (Figure 9C). Importantly, HUH-wt and HUH-PAS revealed no tube formation potential, independently of pre-culture conditions. Obviously, initiation of differentiation only took place in an environment, which was characterized by limitation of oxygen supply and simultaneous diffusion limitation of paracrine and autocrine factors. In recent studies, the phenomena of tumor cell tube formation on matrigel was detected e.g. for glioblastoma, breast cancer [24] and multiple myeloma [25]. In several studies the feature of tube formation was independent from the expression of endothelial markers (vascular mimicry) [26]. However, in the case of HUH-REISO cells, tube formation was associated with induction of the endothelial genes PECAM-1/CD31 and ICAM-2 (Figure 9K/L). In contrast, other endothelial marker genes as vWF, VEGFR2 and VE-Cadherin (data not shown) were neither expressed, nor regulated. Expression of endothelial genes was published by Bussolati *et al.* and Bruno *et al.*, who detected induction of endothelial marker expression, derived from tumor initiating cells (tumor stem cells) by treatment via VEGF [27] or after xenografting [28]. Apparently, HUH-REISO cells, or at least a subpopulation acquired a reprogrammed status characterized by enormous plasticity. This pool of cells reacted on environmental requirements by initiation of differentiation in specialized cells to maintain a balanced tumor microenvironment, ensured sufficient oxygen and nutrient supply and finally counteracted metronomic therapy.

## Conclusions

In conclusion, studying the escape mechanism towards metronomically applied CPA therapy in a HCC xenograft model revealed a multistep process, going beyond unspecific enrichment of a certain subpopulation by the *in vivo* tumor environment. First step of this process was the enrichment of detoxicating enzyme ALDH-1 in combination with upregulation of the Notch1-pathway and its protective downstream effector proteins. Second step was a selection process of pluripotent, stem cell marker expressing cells. These cells increased endothelial transdifferentiation *in vivo* and *in vitro*. Functionality of such endothelial-like cells helped resistant tumors to overcome anti-angiogenic therapy and was the most important finding of our study. Further examinations how modification of Notch signalling may impact cell plasticity and differentiation remain to be explored in future.

## Competing interests

The authors declare that they have no competing interests

## Authors’ contributions

MH carried out the blood flow assays and HE stains. MG carried responsibility for the design and coordination of the study, carried out the first implantation study, the in vitro sensitivity study, partially implemented immunohistochemical stains and helped to draft the manuscript. CM carried out all other experiments, participated in the study design and drafted the manuscript. EW beared responsibility for the conception and coordination of the study, participated in its design and helped to draft the manuscript. All authors read and approved the final manuscript.

## Pre-publication history

The pre-publication history for this paper can be accessed here:

http://www.biomedcentral.com/1471-2407/13/176/prepub

## Supplementary Material

Additional file 1: Table S1 Abbreviations of cell lines and their origin. Click here for file

Additional file 2: Table S2 Used antibodies for immunohistochemistry. Click here for file

Additional file 3: Table S3 Used pairs of primer for qRT-PCR. Click here for file

Additional file 4: Figure S1 *In vitro* control of new generated cell lines. HUH-wt (A), HUH-PAS (B) and HUH-REISO (C) showed no differences in morphology by transmitted light microscopy. Moreover, in FACS analysis cells showed human origin as all cells of HUH-wt (D), HUH-PAS (E) and HUH-REISO (F) were positive in the staining for human epidermal growth factor receptor (hEGF-R), indicated by the shifted red curve in comparison to the correspondent control antibody (white curve). Click here for file

Additional file 5: Figure S3 Hoechst-blood-flow comparison of HUH-REISO vs HUH-PAS after therapy. Intratumoral functional blood flow was visualized by intravenous application of Hoechst 33258 dye (blue). Cryosections (5μm) of HUH-PAS control tumors after 2×CPA treatment (A-C) showed a clear diminished functional blood flow in comparison to treated HUH-REISO tumors (D-F). To ensure to have tumor-material for analysis, the beginning of treatment was late for HUH-PAS tumors, when tumors reached an average volume of 254 mm^3^. Click here for file

Additional file 6: Figure S2 Software based analysis of Matrigel assay. The software based analysis system projected a mask on pictures from transmitted light microscopy of matrigel assay (same pictures as described in Figure 9D, E and F). In white are confluent areas, in blue are nodular structures coloured and built counted tubes with orange and red coloured areas. The tube skeleton is indicated in the thin white lines and their crossing points are defined as branching points. As HUH-wt (A) and HUH-PAS (B) revealed no tube formation, the projected mask is hardly coloured (D and E). HUH-REISO (C) showed low amount of confluent white area and accordingly contained all other aspects of functional tube formation in the mask (F). Click here for file
