# Supplementary material for: Characterization of in vivo chemoresistant human hepatocellular carcinoma cells with transendothelial differentiation capacities
Source: BMC Cancer. 2013 Apr 2;13:176. doi: 10.1186/1471-2407-13-176 (PMC3626554; doi:10.1186/1471-2407-13-176)
Supplement: Additional file 4: Figure S1 — In vitro control of new generated cell lines. HUH-wt (A), HUH-PAS (B) and HUH-REISO (C) showed no differences in morphology by transmitted light microscopy. Moreover, in FACS analysis cells showed human origin as all cells of HUH-wt (D), HUH-PAS (E) and HUH-REISO (F) were positive in the staining for human epidermal growth factor receptor (hEGF-R), indicated by the shifted red curve in comparison to the correspondent control antibody (white curve). [file 1471-2407-13-176-S4.pdf]

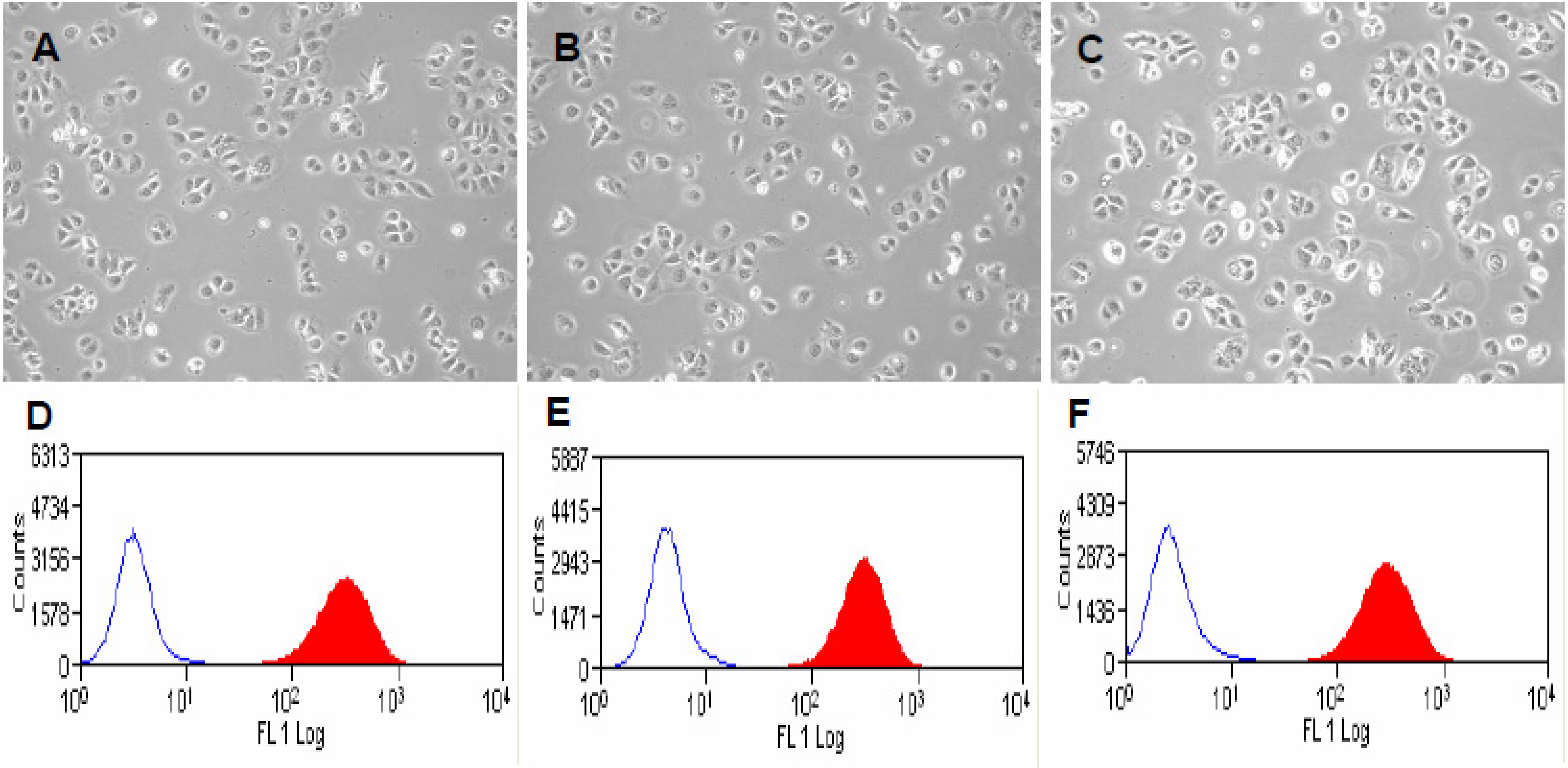

Supplementary Figure 1 – *in vitro* control of new generated cell lines. HUH-wt (A), HUH-PAS (B) and HUH-REISO (C) showed no differences in morphology by transmitted light microscopy. Moreover, in FACS analysis cells showed human origin as all cells of HUH-wt (D), HUH-PAS (E) and HUH-REISO (F) were positive in the staining for human epidermal growth factor receptor (hEGF-R), indicated by the shifted red curve in comparison to the correspondent control antibody (white curve).
